# Early decline in left ventricular ejection fraction predicts doxorubicin cardiotoxicity in lymphoma patients

**DOI:** 10.1038/sj.bjc.6600346

**Published:** 2002-06-05

**Authors:** T Nousiainen, E Jantunen, E Vanninen, J Hartikainen

**Affiliations:** Department of Medicine, Kuopio University Hospital and University of Kuopio, FIN 70211 Kuopio, Finland; Department of Clinical Physiology and Nuclear Medicine, Kuopio University Hospital and University of Kuopio, FIN 70211 Kuopio, Finland

**Keywords:** doxorubicin, cardiotoxicity, radionuclide ventriculography

## Abstract

Thirty adult patients with non-Hodgkin's lymphoma were studied to evaluate prospectively the significance of early decline in left ventricular ejection fraction after low cumulative doxorubicin dose (200 mg m^−2^) in predicting the later impairment of left ventricular function. Cardiac function was monitored with radionuclide ventriculography at baseline and after cumulative doxorubicin doses of 200, 400 and 500 mg m^−2^. Cardiotoxicity was defined as a decrease in left ventricular ejection fraction of more than 10% units to a final left ventricular ejection fraction ⩽50%. Twenty-eight patients received doxorubicin ⩾400 mg m^−2^ and were evaluable for cardiotoxicity. Clinical heart failure developed in two patients (7%) after a cumulative doxorubicin dose of 500 mg m^−2^. Left ventricular ejection fraction decreased more than 10% absolute ejection fraction units to a final left ventricular ejection fraction ⩽50% in 10 patients (36%). Left ventricular ejection fraction decreased from 56±1.5% to 53.6±1.5% (*P*=0.016) in patients with no cardiotoxicity, and from 60.8±2.4% to 41.8±2.0% (*P*<0.001) in patients with cardiotoxicity. For patients who developed cardiotoxicity, the fall in left ventricular ejection fraction after a cumulative doxorubicin dose of only 200 mg m^−2^ was highly significant (left ventricular ejection fraction 49.7±1.8%, *P*=0.001 *vs* baseline). In receiver operator characteristic analysis, the area under the curve for the decrease in left ventricular ejection fraction at a cumulative doxorubicin dose of 200 mg m^−2^ for predicting cardiotoxicity in all patients was 0.858. The decrease in left ventricular ejection fraction of more than 4% units after a cumulative doxorubicin dose of 200 mg m^−2^ had a 90% sensitivity and 72% specificity for predicting later cardiotoxicity. Our results show that the significant impairment of left ventricular function during doxorubicin therapy can be predicted early, already at low cumulative doxorubicin doses. This finding may be of value in identifying patients at high or low risk for the development of anthracycline cardiotoxicity.

*British Journal of Cancer* (2002) **86**, 1697–1700. doi:10.1038/sj.bjc.6600346 www.bjcancer.com

© 2002 Cancer Research UK

## 

Doxorubicin is one of the most potent anti-neoplastic agents in the treatment of lymphoid malignancies and many solid tumours. However, its therapeutic value is limited by cumulative dose-related cardiotoxicity. The incidence of congestive heart failure (CHF) during doxorubicin treatment has been reported to be 3% at a dose of 400 mg m^−2^ and 7% at a dose of 550 mg m^−2^ (von Hoff *et al*, 1979), but the occurrence of CHF is unpredictable. Attempts to prevent CHF include empiric dose limitation and serial assessment of left ventricular function. Only endomyocardial biopsy has been considered to be sensitive and specific enough in predicting the development of CHF (Mason *et al*, 1978; Billingham and Bristow, 1984), but the invasiveness and potential complications of the procedure limit its clinical use. Radionuclide ventriculography (RVG) has been regarded as the best noninvasive method in identifying subclinical anthracycline cardiotoxicity in adult patients (Alexander *et al*, 1979; Schwartz *et al*, 1987; Ganz *et al*, 1996). Guidelines based on changes in systolic and diastolic left ventricular function have been given for monitoring patients receiving anthracycline therapy (Alexander *et al*, 1979; Schwartz *et al*, 1987; Ganz *et al*, 1996).

Despite the introduction of new imaging techniques like Indium-111-antimyosin (Carrio *et al*, 1995; Maini *et al*, 1997) and Iodine-123-metaiodobenzylguanidine (MIBG) scans (Valdés Olmos *et al*, 1995; Carrio *et al*, 1995) and serum markers of left ventricular dysfunction like natriuretic peptides (Bauch *et al*, 1992; Nousiainen *et al*, 1999; Meinardi *et al*, 2001) for the monitoring of cardiac function during anthracycline therapy, none of these methods has been able to solve the problem of early detection of severe anthracycline-induced cardiotoxicity.

In the present study we investigated the significance of early impairment of cardiac systolic function after low cumulative doxorubicin dose (200 mg m^−2^) and its ability to predict the later decrease in left ventricular ejection fraction (LVEF) during doxorubicin therapy.

## MATERIALS AND METHODS

### Patients

Thirty consecutive adult patients ⩽75 years of age with previously untreated non-Hodgkin's lymphoma, who were scheduled to receive CHOP chemotherapy, were studied. The patients were regarded as eligible for study entry if they had not received prior anthracycline therapy or radiation therapy to mediastinum. A history of heart failure was also considered as an exclusion criterion. Two patients died early during the treatment due to progressive lymphoma and were not evaluable. Thus, the final study population consisted of 28 patients (17 men and 11 women) with a mean age of 53 years (range 22–75 years). Six patients (21%) were ⩾65 years. Six patients (21%) had a pre-existing cardiovascular disease (four patients had WHO class II hypertension, one patient had suffered from a prior myocardial infarction and one patient from recurrent episodes of atrial fibrillation). Of the patients with a prior cardiovascular disease, two patients were over 65 years of age.

Approval for the study was obtained from the local ethical committee and the patients provided written informed consent.

### Chemotherapy

The CHOP chemotherapy was administered in standard doses (cyclophosphamide, 750, doxorubicin, 50 and vincristine 1.4 mg m^−2^ were given intravenously on day 1 and prednisolone 100 mg orally on days 1–5). Doxorubicin was given as a 30 min infusion. The cycle was repeated every 3 weeks to a total of 10 cycles. Doxorubicin was discontinued if left ventricular ejection fraction (LVEF) decreased below 45% (Druck *et al*, 1984; Ganz *et al*, 1996). No radiotherapy was given during the study period.

### Radionuclide ventriculography

Radionuclide ventriculography (RVG) was performed at baseline and after a cumulative doxorubicin dose of 200, 400 and 500 mg m^−2^. Left ventricular ejection fraction (LVEF) was assessed using standard techniques (Wackers *et al*, 1979). The equilibrium RVG was performed with semi *in vitro* technetium-99m-labelled blood cells (injected activity 670 MBq). A large field-of-view gamma camera equipped with a high-sensitivity parallel hole collimator was used for imaging. The cardiac cycle was divided into 24 frames with a 10% tolerance. Ten million counts were acquired. Data were analysed with a commercial cardiac software (MGQ, Nuclear Diagnostics Ab, Hägersten, Sweden). If the LVEF decreased more than 10% units or was <50%, the RVG scan was repeated before the subsequent CHOP course. A decrease of LVEF >10% units to an final LVEF ⩽50% was used as a cut-off point in the analyses as indicative of doxorubicin-induced cardiotoxicity (CT) (Schwartz *et al*, 1987).

### Statistical methods

All calculations were performed with SPSS/PC statistical program (version 9.0, SPSS Inc., Chicago IL, USA). The differences for continuous variables over time were analysed using general linear model for repeated measures. Paired, two tailed *t*-tests were applied for *post-hoc* analyses. Additional subgroup analyses for patients with and without left ventricular dysfunction as defined on the basis of a decrease in LVEF were performed using Mann–Whitney *U*-test for continuous variables and with Chi-Square test for nominal data. Receiver operator characteristic analysis (ROC) was used to evaluate the diagnostic ability of the decrease in LVEF after a cumulative doxorubicin dose of 200 mg m^−2^ to predict the development of doxorubicin-induced cardiotoxicity (CT). A *P*-value <0.05 was considered as statistically significant. The data are expressed as mean±s.e.

## RESULTS

Twenty-eight patients of the initial patient population received at least eight courses of CHOP (cumulative doxorubicin dose ⩾400 mg m^−2^) and could be used in the analysis for evaluating cardiotoxicity. Two patients died early because of progressive lymphoma. Twenty-four patients received 10 cycles (cumulative doxorubicin dose 500 mg m^−2^). The reason for discontinuation or change of the treatment in four patients after eight cycles of CHOP was suboptimal treatment response or disease progression.

**Figure 1:**
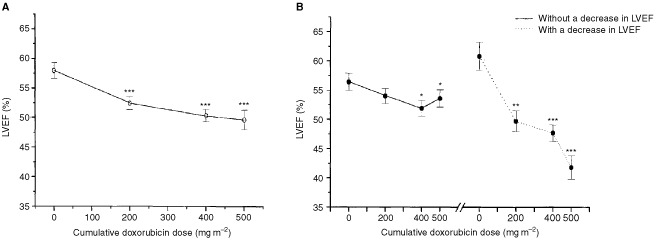
Changes in left ventricular ejection (LVEF) during doxorubicin therapy. (**A**) represents data of all patients, and (**B**) represents data without and with the decrease in LVEF ⩾10% units to the final LVEF ⩽50%. *P<0.05, ** *P*<0.01, ***P<0.001 *vs* baseline.

The baseline LVEF of the patients was 58.0±1.3%. It decreased to 52.5±1.1% (*P*<0.001), 50.4±1.0% (*P*<0.001) and 49.6±1.7% (*P*<0.001) after cumulative doxorubicin doses of 200, 400 and 500 mg m^−2^, respectively (Figure 1A

). LVEF decreased more than 10% absolute EF units to a final LVEF ⩽50% in 10 patients (36%). This cut-off point was reached after a cumulative dose of 200 mg m^−2^ in two patients, after 400 mg m^−2^ in four patients and after 500 mg m^−2^ in four patients. Clinical heart failure developed in two patients (7%) at 1 month and 10 months after the last dose of doxorubicin (cumulative dose 500 mg m^−2^). The patients were a 43-year old previously healthy man and a 70-year-old man with no history of heart disease or hypertension. These patients presented with the decrease in LVEF of more of 10% units and ⩽50% after a cumulative doxorubicin doses of 400 and 500 mg m^−2^, respectively.

### Comparison of patients with and without cardiotoxicity

**Figure 2:**
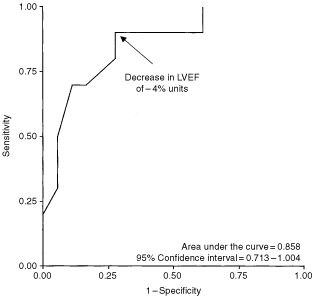
ROC plot of the sensitivity and specificity of the decrease in left ventricular ejection fraction (LVEF) after a cumulative doxorubicin dose of 200 mg m^−2^ to predict the decrease in LVEF ⩾10% units to the final LVEF ⩽50% during doxorubicin therapy. The arrow indicates the decrease in LVEF of −4% units.

There were no differences in age, gender, hypertension, the use of β-adrenoceptor blocking drugs, or LVEF between the patients with or without CT, at baseline. In patients with no CT, LVEF decreased from 56.4±1.5% to 54.0±1.3% (*P*=ns), 51.9±1.2% (*P*=0.029) and to 53.6±1.5% (*P*=0.016), whereas in patients with CT, LVEF decreased from 60.8±2.4% to 49.7±1.8% (*P*=0.001), 47.7±1.4% (*P*<0.001) and to 41.8±2.0% (*P*<0.001) after cumulative doxorubicin doses of 200, 400 and 500 mg m^−2^, respectively (Figure 1B). There was a statistically significant difference in the decrease in LVEF in patients with and without CT already after a cumulative doxorubicin dose of 200 mg m^−2^ (−11.1±2.3% units *vs* −2.4±1.2% units, *P*=0.001). The accuracy of the decrease in LVEF after a cumulative doxorubicin dose of 200 mg m^−2^ to predict the decrease in LVEF >10% units to the final LVEF ⩽50% in all patients was estimated with ROC analysis. The area under the curve was 0.858 (95% CI=0.713–1.004) (Figure 2

). The decrease in LVEF of more than 4% after a cumulative doxorubicin dose of 200 mg m^−2^ units had a 90% sensitivity and 72% specificity for predicting CT. This decrease in LVEF had a positive predictive value of 64% and the negative predictive value of 93% for the development of CT. Both patients with clinical CHF after doxorubicin therapy had a decrease in LVEF of 8% units after a cumulative doxorubicin dose of 200 mg m^−2^.

## DISCUSSION

Early detection of subclinical anthracycline cardiotoxicity, and ultimately the prevention of clinical congestive heart failure, is a continuing challenge in clinical oncology. Attempts to minimise cardiotoxicity include serial monitoring of cardiac function or empiric anthracycline dose limitation. However, empiric limitation or modification of anthracycline dose, e.g. by risk factors, pose a risk of premature discontinuation of effective anthracycline therapy. On the other hand, because of a wide individual variability in toxic anthracycline doses (von Hoff *et al*, 1979; Schwartz *et al*, 1987), cardiotoxicity may occur at unexpectedly low cumulative doses. Serial assessment of LVEF by RVG has been the most commonly used method of cardiac monitoring. According to the guidelines proposed by Schwartz *et al* (1987), doxorubicin therapy should be discontinued, if LVEF decreases >10% units to a final LVEF ⩽50%. A baseline study should be performed before the administration of cumulative doxorubicin dose of 100 mg m^−2^, the next LVEF measurement should be performed at 250–300 mg m^−2^ and again after 450 mg m^−2^ (after 400 mg m^−2^, if there are risk factors) and then after every treatment course (Schwartz *et al*, 1987). However, low dose cardiotoxicity can occur especially in patients with pre-existing risk factors and predispose the patients to irreversible cardiac damage.

In this prospective study, we evaluated the importance of low doxorubicin dose cardiotoxicity and found that in patients, with the ultimate cardiotoxic decrease in LVEF as proposed by Schwartz *et al* (1987), the impairment of LVEF was statistically significant as early as after a cumulative doxorubicin dose of 200 mg m^−2^. Furthermore, we observed that the decrease in LVEF of more than 4% units from the baseline after a cumulative doxorubicin dose of 200 mg m^−2^ could in a specific and sensitive manner predict the later decrease in LVEF. Thirty-six per cent of our patients had a decrease in LVEF of more of 10% units and ⩽50% after doxorubicin therapy. However, only two patients developed manifest CHF after completion of therapy (cumulative doxorubicin dose of 500 mg m^−2^). Both patients had a decrease in LVEF of 8% units after a cumulative doxorubicin dose of 200 mg m^−2^.

In this study, the doxorubicin cardiotoxicity was defined as a decrease in LVEF (Schwartz *et al*, 1987) due to the small number of patients studied and thus with low incidence of clinical CHF. Although the guideline decrease in LVEF as proposed by Schwartz *et al* (1987) is based on retrospective analysis, the risk of CHF was reduced by four-fold when the guidelines were followed in their study. Only two out of 70 patients (3%) in the cohort whose management was concordant with the guidelines developed mild CHF (Schwartz *et al*, 1987). Our observation of the value of the early decrease in LVEF after a cumulative doxorubicin dose of 200 mg m^−2^ in predicting this guideline decrease in LV is intriguing and may allow the identification of high risk patients, who might need a more careful monitoring earlier in their treatment course. On the other hand, the high negative predictive value of 93% of the early decrease in LVEF for the later impairment of LV function seems to identify the patients with better tolerance to higher cumulative anthracycline doses and hence less need for cardiac monitoring. However, it must be born in mind that the small number of the patients in our study might have had impact on the results of ROC-analysis. Furthermore, these results are preliminary and need to be confirmed in larger prospective studies using clinically confirmed CHF as an endpoint.

In order to prevent anthracycline cardiotoxicity, attempts have focused on the development of safer anthracycline derivates like liposomal doxorubicin (Alberts and Garcia, 1997) or molecules with cardioprotective effects (Swain, 1998). On the other hand, new sophisticated methods have been investigated for early detection and prevention of anthracycline-induced cardiotoxicity. Indium-111-antimyosin scintigraphy has proven out to be a sensitive indicator of myocardial cell injury including anthracycline toxicity (Carrio *et al*, 1995; Maini *et al*, 1997). However, the antibody is not commercially available at the moment. Iodine-123-metaiodobenzylguanidine (MIBG) is a norepinephrine analogue which is taken up by myocardial sympathetic nerve endings and reflects myocardial adrenergic integrity or function (Wieland *et al*, 1981; Sisson *et al*, 1987). Decreased cardiac MIBG uptake has been reported in some studies to precede the decrease in LVEF in patients treated with doxorubicin (Valdés Olmos *et al*, 1995; Carrio *et al*, 1995). However, MIBG scintigraphy is available only in few centers and the method cannot be regarded as a standard method of follow-up of anthracycline therapy.

Plasma natriuretic peptides are biochemical markers of left ventricular dysfunction. Atrial natriuretic peptide (ANP) and brain natriuretic peptide (BNP) are synthesised, stored and secreted from the heart in response to atrial or ventricular overload (Ruskoaho, 1992; Levin *et al*, 1998). Elevated levels of plasma natriuretic peptides have been reported in patients treated with anthracyclines (Bauch *et al*, 1992; Nousiainen *et al*, 1999; Meinardi *et al*, 2001). Furthermore, it has been suggested that the measurement of plasma natriuretic peptides could be used as an early indicator of anthracycline cardiotoxicity (Bauch *et al*, 1992; Meinardi *et al*, 2001). However, we have previously shown that the increased secretion of natriuretic peptides during doxorubicin therapy is a compensatory phenomenon, not preceding but following the decrease in LVEF (Nousiainen *et al*, 1999).

Thus, optimising the anthracycline dose and minimising the risk of CHF; a dilemma in 1987 (Schwartz *et al*, 1987) is still a dilemma today. In spite of the various methods for monitoring anthracycline cardiotoxicity, none of the non-invasive methods have proven out to be sensitive and specific enough to predict the development of cardiomyopathy. Furthermore, despite the use of cardioprotectors and new anthracycline derivates like liposomal doxorubicin, monitoring of cardiac function is still needed in clinical practice. In this study, we observed that a significant decrease in LVEF during doxorubicin treatment can be observed, and later deterioration of LV function predicted early, at a very low cumulative doxorubicin dose.

Although our findings are preliminary, they may offer a more rational and individualised basis for cardiac follow-up of patients receiving anthracycline-based chemotherapy. In particular, patients with known risk factors for anthracycline-induced cardiotoxicity, like older age, pre-existing cardiovascular disease or history of mediastinal radiotherapy might be candidates for this early monitoring, which quite accurately predicted the future impairment of left ventricular function in this study. On the other hand, patients with no significant decrease in LVEF at a cumulative doxorubicin dose of 200 mg m^−2^, seem to have minor risk of cardiotoxicity and thus perhaps less need for cardiac monitoring.
